# “Monoclonal-Type” Plastic Antibodies for COVID-19 Treatment: What Is the Idea?

**DOI:** 10.3390/jfb11020043

**Published:** 2020-06-17

**Authors:** Francesco Puoci

**Affiliations:** 1 Health and Nutritional Sciences, Department of Pharmacy, University of Calabria, 87036 Rende (CS), Italy; francesco.puoci@unical.it; Tel.: +39-0984-493151; 2 Macrofarm s.r.l., Health and Nutrition Sciences, c/o Department of Pharmacy, University of Calabria, 87036 Rende (CS), Italy

In late December 2019, an outbreak due to a novel coronavirus, initially called 2019-nCoV, was reported in Wuhan, China [1]. This newly discovered etiological agent is now known as SARS-CoV-2 (Severe Acute Respiratory Syndrome Coronavirus 2) and is responsible for COVID-19, which resulted in a pandemic. As of 27 May 2020, indeed, 5,488,825 cases and 349,095 deaths have been globally reported [2].

Currently, there are no approved drugs or vaccines for the treatment and prevention of COVID-19; therefore, in the absence of effective therapeutics, different strategies are being explored. One of these is represented by the evaluation of the efficacy of repurposed drugs, used individually or in combination, to counteract the virus infection and/or improve clinical symptoms in severe patients [3]. Another approach, which is receiving considerable attention, is the development of monoclonal antibodies able to target susceptible sites on viral surface proteins blocking the infection process [4]. However, traditional monoclonal antibodies present some functional drawbacks, which limit their extensive use as therapeutic agents [5]. Monoclonal antibodies, indeed, are very expensive to produce and are characterized by a restricted stability, [6] unsuitable pharmacokinetics and tissue penetration and impaired interactions with the immune system [5].

In the aim to overcome these drawbacks, a very promising alternative to traditional antibodies is represented by plastic antibodies made by polymeric biomaterials. In this context, Molecular Imprinting is an interesting and powerful technology for the development of “monoclonal-type” plastic antibodies based on Molecularly Imprinted Polymers (MIPs). These polymeric materials, indeed, are characterized by specific and selective recognition properties for a target molecule called a template [7]. The synthesis of MIPs involves the polymerization of functional and crosslinking monomers around the chosen template, which is then extracted, resulting in a porous polymeric network characterized by the presence of binding cavities fitting the size, shape and functionalities of the target compound.

As they are synthetic materials, MIPs are robust, physically and chemically stable in a wide range of conditions and more easily available due to their low-cost, reproducibility and relatively fast and easy preparation compared to the biological counterpart. Given these characteristics, MIPs can represent a valid alternative to conventional antibodies.

In literature, several studies report on the preparation of MIPs for proteins and other biomacromolecules detection. Wang et al. developed a fluorescent nanosensor for the detection of ovalbumin, which was used as a glycoprotein model [8]. The ratiometric nanosensor was obtained by the combination of blue color carbon dots (CDs), not involved in the imprinting process, and green color core-shell imprinted polymers synthesized by post-imprinting and using fluorescein isothiocyanate (FITC) as a fluorescence probe. In another study, a label-free sensor for the detection of fibrinopeptide B (FPB) in urine, a biomarker of venous thromboembolism, was obtained combining photonic crystals and molecularly imprinted polymers [9]. The resulting sensor exhibited optical properties that change upon detection of low concentrations of the target compound in urine. Protein sensors based on electroactive MIPs were also fabricated by Zhao et al. employing bovine serum albumin and trypsin as model templates and a linear electro-polymerizable molecularly imprinted polymer as a macromonomer [10].

Some recent studies report the development of MIPs-based sensors for the selective detection of viruses such as Japanese Encephalitis Virus (JEV) and Hepatitis A Virus (HAV) through the Resonance Light Scattering (RLS) technique. In the first work, [11] a magnetic surface molecularly imprinted-resonance light scattering sensor was prepared using Fe_3_O_4_ microspheres coated by silicon as imprinting substrates and aminopropyl-triethoxysilane (APTES) as functional monomers for fixing JEV through a polymerization process of tetraethyl-orthosilicate (TEOS). In the second one [12], molecular imprinting resonance light scattering nanoprobes able to selectively bind HAV were fabricated using pH-responsive metal-organic frameworks.

Most of the research studies on MIPs for biomacromolecules, such as proteins and viruses, are focused on the preparation of sensors and probes for the detection of these targets, while only a few works are devoted to the therapeutic use of these polymeric materials. 

One example is given by Xu et al. [13], who presented molecularly imprinted polymer nanoparticles able to bind the highly conserved and specific peptide motif SWSNKS (3S), an epitope of the envelope glycoprotein 41 (gp41) of human immunodeficiency virus type 1 (HIV-1). The imprinted nanoparticles were produced by solid-phase synthesis and could find a potential application as artificial antibodies for immunoprotection against HIV.

At this time, Parisi et al. at the Department of Pharmacy, Health and Nutritional Sciences of the University of Calabria, are developing “monoclonal-type” plastic antibodies based on MIPs able to selectively bind a portion of SARS-CoV-2 spike protein to block its function and, thus, the infection process (Figure 1) [14].

The coronavirus spike protein is a surface protein that mediates host recognition and attachment. It consists of two functional subunits: the S1 subunit which contains a receptor-binding domain (RBD) responsible for host cell receptor recognizing and binding, and the S2 subunit which is involved in the fusion of the viral and host membranes [15]. The spike protein, thus, represents the common and primary target for the development of antibodies, vaccines and therapeutic agents.

Therefore, polymeric imprinted nanoparticles could be potentially used as drug-free therapeutics in the treatment of the SARS-CoV-2 infection. Plastic antibodies targeting vulnerable sites on viral surface proteins, indeed, could disable receptor interactions and protect an uninfected host that is exposed to the virus. In vivo applications demand MIPs in the form of nanoparticles and there are evidences that nanoMIPs are not toxic in cell culture or when tested with mice [16].

Moreover, when loaded with antiviral agents, these nanoparticles could act as a powerful multimodal system combining their ability to block the virus spike protein with the targeted delivery of the loaded drug. In addition, the same nanoparticles can be further engineered to become an immunoprotective vaccine or an MIP-based sensor for diagnostic purpose.

Based on these considerations, Molecular Imprinting represents a very promising technology for the preparation of polymeric materials with high selective recognition abilities for a target molecule. On the other hand, the imprinting of biomacromolecules, including peptides, proteins, whole viruses or parts of them, presents several challenges due to the size, solubility, fragile structure and stability of these templates. Moreover, virus and viral components availability is also a key issue. Last but not least, sensitivity and selectivity of these polymeric matrices require further improvement to be comparable to those of natural antibodies. 

The research work of Parisi et al. aims to overcome these limits to obtain MIP nanoparticles able to selectively recognize and bind the spike protein of the novel coronavirus and counteract the infection process.

## Figures and Tables

**Figure 1:**
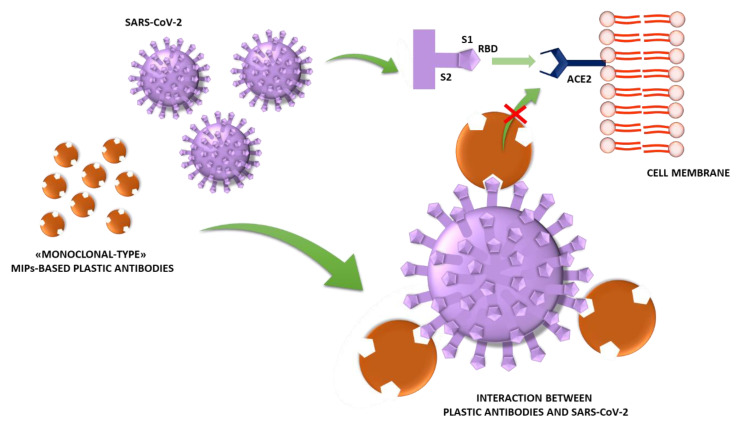
Schematic representation of the interaction between Molecularly Imprinted Polymers (MIP)-based “monoclonal-type” plastic antibodies and SARS-CoV-2 (Severe Acute Respiratory Syndrome Coronavirus 2).
